# Toward Optimized Charge Transport in Multilayer Reduced Graphene Oxides

**DOI:** 10.1021/acs.nanolett.1c03883

**Published:** 2022-03-01

**Authors:** Mustafa Neşet Çınar, Aleandro Antidormi, Viet-Hung Nguyen, Alessandro Kovtun, Samuel Lara-Avila, Andrea Liscio, Jean-Christophe Charlier, Stephan Roche, Hâldun Sevinçli

**Affiliations:** † Department of Materials Science and Engineering, Izmir Institute of Technology, 35430 Urla, Izmir, Turkey; ‡ Catalan Institute of Nanoscience and Nanotechnology, CSIC and The Barcelona Institute of Science and Technology, Campus UAB, 08193 Bellaterra, Barcelona, Spain; ¶ Institute of Condensed Matter and Nanosciences, Université catholique de Louvain (UCLouvain), B-1348 Louvain-la-Neuve, Belgium; § Consiglio Nazionale delle Ricerche, Istituto per la Sintesi Organica e la Fotoreattivitá, (CNR-ISOF), via Gobetti 101, 40129 Bologna, Italy; ∥ Department of Microtechnology and Nanoscience, Chalmers University of Technology, Kemivägen 9, 41296 Gothenburg, Sweden; ⊥ Consiglio Nazionale delle Ricerche, Istituto per la Microelettronica e Microsistemi, Roma Unit (CNR-IMM), via del fosso del cavaliere 100, 00133 Rome, Italy; # ICREA−Institució Catalana de Recerca i Estudis Avançats, 08010 Barcelona, Spain

**Keywords:** disordered van der Waals thin films, reduced graphene oxides, charge transport, quantum transport, interlayer transport, multilayer transport scaling law

## Abstract

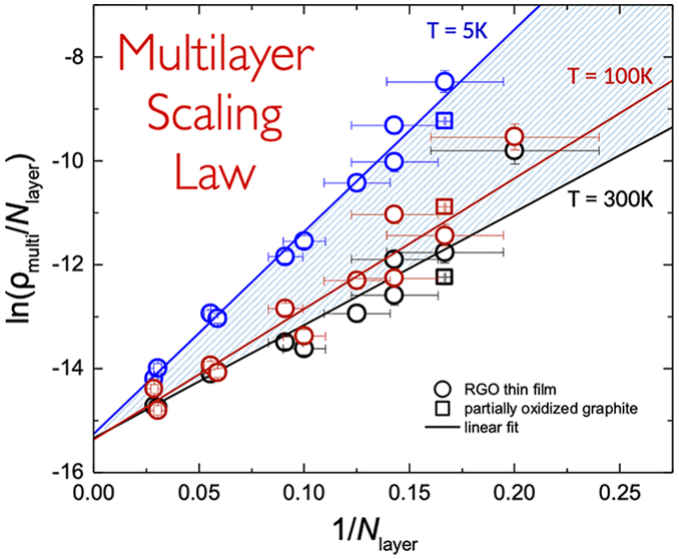

In the context of graphene-based composite applications, a complete understanding of charge conduction in multilayer reduced graphene oxides (rGO) is highly desirable. However, these rGO compounds are characterized by multiple and different sources of disorder depending on the chemical method used for their synthesis. Most importantly, the precise role of interlayer interaction in promoting or jeopardizing electronic flow remains unclear. Here, thanks to the development of a multiscale computational approach combining first-principles calculations with large-scale transport simulations, the transport scaling laws in multilayer rGO are unraveled, explaining why diffusion worsens with increasing film thickness. In contrast, contacted films are found to exhibit an opposite trend when the mean free path becomes shorter than the channel length, since conduction becomes predominantly driven by interlayer hopping. These predictions are favorably compared with experimental data and open a road toward the optimization of graphene-based composites with improved electrical conduction.

Understanding charge transport in multilayered van der Waals materials has become an attractive and challenging problem, in the perspective of both fundamental and applied research on graphene-based composites.^1,2,35^ Indeed, graphene-related materials (including chemically disordered graphene like rGO) have shown remarkable capability to improve charge and thermal conductivities of many insulating flexible materials such as organic polymers, suggesting them as the privileged filler material to reinforce, diversify, and improve the properties and performances of traditional materials used in wearables, flexible electronics, conducting textiles, and thermoplastics. However, accurate understanding of the microscopic mechanisms leading to transport in these complex systems is still a matter of debate. Indeed, recent experimental studies^3−5^ suggested the key role played by various types of defects, as well as interlayer interaction in the transport mechanisms taking place inside the graphene-based composites.

More specifically, transport characteristics in multilayered rGO^5^ were observed to change from a conventional Efros-Shkloskii variable range hopping (ES-VRH)^36,37^ to a temperature-dependent power-law regime, with increasing number of stacked layers. Such findings do not apparently depend on any length scale of the system, since they are observed in both micrometric networks of few nanosheets partially overlapping, as well as in centimeter-scale thin films built from billions of rGO nanosheets randomly stacked. Besides, the resulting localization length is also found to increase (by 3 orders of magnitude) with both the aromatic content and the thickness of the thin films as well, while being roughly independent of the lateral size nanosheet. Accordingly, the main contribution to transport properties in multilayered rGO with random stacking likely stems from a bulk contribution, with marginal nanosheet edge effects.^5^

Here, we investigate charge transport in rGO thin films using state-of-the-art modeling techniques and compared the results with experimental measurements. The multilayered rGO models are constructed using a rectangular ribbon-like geometry with a width of 20 nm and periodic boundary conditions in the transverse direction. Disorder is introduced by incorporating random distributions of chemical defects (Figure 1a) such as divacancies (0.29%), Stone–Wales defects (0.01%), and epoxides (4.7%). Such chemical nature and densities of defects have been extracted from atomistic samples of rGO obtained by classical molecular simulations of the thermal reduction process of graphene oxide (GO) sheets.^6^ More specifically, to produce the models, we employ the thermal annealing protocol described in ref (6), since it reproduces the main structural features observed in measured rGO samples. In these MD simulations, large-scale GO samples with an initial equivalent concentration of epoxide and hydroxyl groups, amounting to a O/C ratio of 35%, were annealed at 900 °C. At the end of the annealing process, the final concentration of oxygen atoms was found to be approximately 5%, in excellent agreement with the experimental results reported in ref (5).

**Figure 1:**
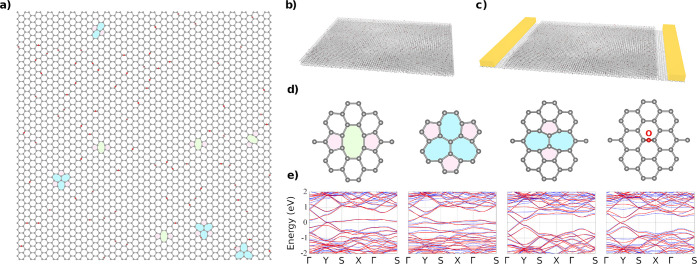
Reduced graphene oxide hosts different types of defects that are randomly distributed (a), where carbon atoms are colored with gray, and oxygen with red. Pentagon, heptagon, and octagons are indicated with red, blue, and green shades. Charge transport calculations in typical multilayered rGO models are investigated in rGO using (b) the Kubo formalism to estimate the bulk electronic conductivity and (c) the Landauer-Büttiker approach in a conventional device geometry. (d) Atomic structures of various incorporated defect types (pristine divacancy (585), reconstructed divacancy (555–777), Stone–Wales (55–77), and epoxy defects) and their corresponding tight-binding models (e) extracted from *ab initio* band-structure calculations.

These chemical defects are known to induce lattice distortion and charge redistribution locally around their spatial location.^7−12^ Since these detrimental effects are local, rGO models with defect concentrations similar to those investigated in this study can be modeled using a conventional tight-binding (TB) Hamiltonian (as for graphene) but with specific adjustments made locally around the defect position. Within such a framework, the Hamiltonian of the rGO system presents a simple form allowing one to further perform large-scale transport calculations in realistic samples containing more than 10^6^ atoms and with varying defect concentrations. The parametrized *p*_*z*_ TB Hamiltonian reads
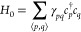
1where in-plane couplings are limited to nearest-neighboring interactions. In contrast with other models in the literature (see refs (13, 14)), the effects of chemical defects are described by properly adjusting the TB parameters to recover *ab initio* results. The optimization of these TB parameters for each specific single defect are obtained by fitting first-principles electronic band structures of the graphene supercell containing a single defect (see Supporting Information).

Importantly, the change in C–C bond length due to the lattice distortion around the defects is accounted for by computing the hopping energies as a function of C–C bond length (*r*_*pq*_) and defining γ_*pq*_ = γ_0_ exp[−β(*r*_*pq*_/*r*_0_ – 1)] with nearest-neighbor hopping energy γ_0_ = −2.6 eV, nearest-neighbor distance *r*_0_ = 1.42 Å, and the decay parameter β = 3.37.^15^ The local doping due to the localized states induced by defects and impurities is also included by modulating onsite energies as a distance-decay function centered at the defect positions. Since the atomic positions are altered around defects, the parametrization for interlayer coupling also requires one to account for changes in interatomic distances compared to Bernal graphite (AB-stacking). Consequently, to determine TB couplings in the presence of varying bond lengths around structural defects, an exponential decay-based model formula is used following γ(*r*) = γ_1_ exp(β_*z*_(1 – *r*/*z*)) with interlayer coupling energy γ_1_ = 0.36 eV, the corresponding decay parameter β_*z*_ = 24.99, and interlayer distance *z* = 3.34 Å.^16,17^ Further details and comparison against density functional theory and other methods are given in the Supporting Information. The different types of considered defects used in the model are illustrated in Figure 1d. The electronic bands obtained from TB parametrization are in excellent agreement with first-principles density functional theory results (Figure 1e). Dependence of electronic structure and transport properties of twisted graphene layers has been studied intensely during the last years, and it was shown that even small twist angles give rise to substantial changes.^18−20,33^ A similar effect is not expected in randomly stacked rGO, because defects are strong scattering centers compared to the weak interlayer coupling. Hence, scattering from impurities is the dominant mechanism for the randomization of carriers’ momenta causing more than 100 scatterings within the interlayer diffusion length. Indeed, conductivity was shown to be practically independent of the twist angle in twisted bilayer graphene in the presence of lower concentrations of random defects.^21^

Kubo-Greenwood (KG) and Landauer-Büttiker (LB) transport formalisms are used to study the electronic conductivity and conductance in multilayered rGO models and for varying transport geometries (see Figure 1b,c, respectively). The LB method^22−24^ allows one to include the charge injection from contact electrodes (Figure 1c), whereas the KG method gives access to bulk properties^25^ (Figure 1b). These two techniques enable contrasting bulk properties with “device” related transport (see Supporting Information for details). Note that neither electron–phonon coupling nor many-body effects are included in the present study.

Figure 2a presents the energy-dependent conductance for the mono-, bi-, and trilayer rGO for identical defect concentration, calculated within the LB approach. When layers are free from defects (pristine graphene), adding new layers will act as a supplemental scattering source, hence reducing the total conductance (see Figure 2a-inset). Indeed, in defect-free monolayer graphene, transport is ballistic and a maximum V-shaped conductance curve is obtained. When more clean layers are stacked on top of this graphene layer, new scattering channels are opened and an interface resistance is formed. Consequently, the conductance of bi- and trilayers (multilayered stack) is reduced below the single layer of pristine monolayer graphene. Actually, such conductance decay is not specific to disorder-free systems but is also observed in low-defect concentrations as well (see Figure S6). Importantly, this reduction of the electronic transmission is found when the electrodes are attached to the bottom layer, and it decreases in a non-monotonic way with the number of added layers. This behavior can be understood as driven by complicated interference processes taking place across the layers and throughout the central region.

**Figure 2:**
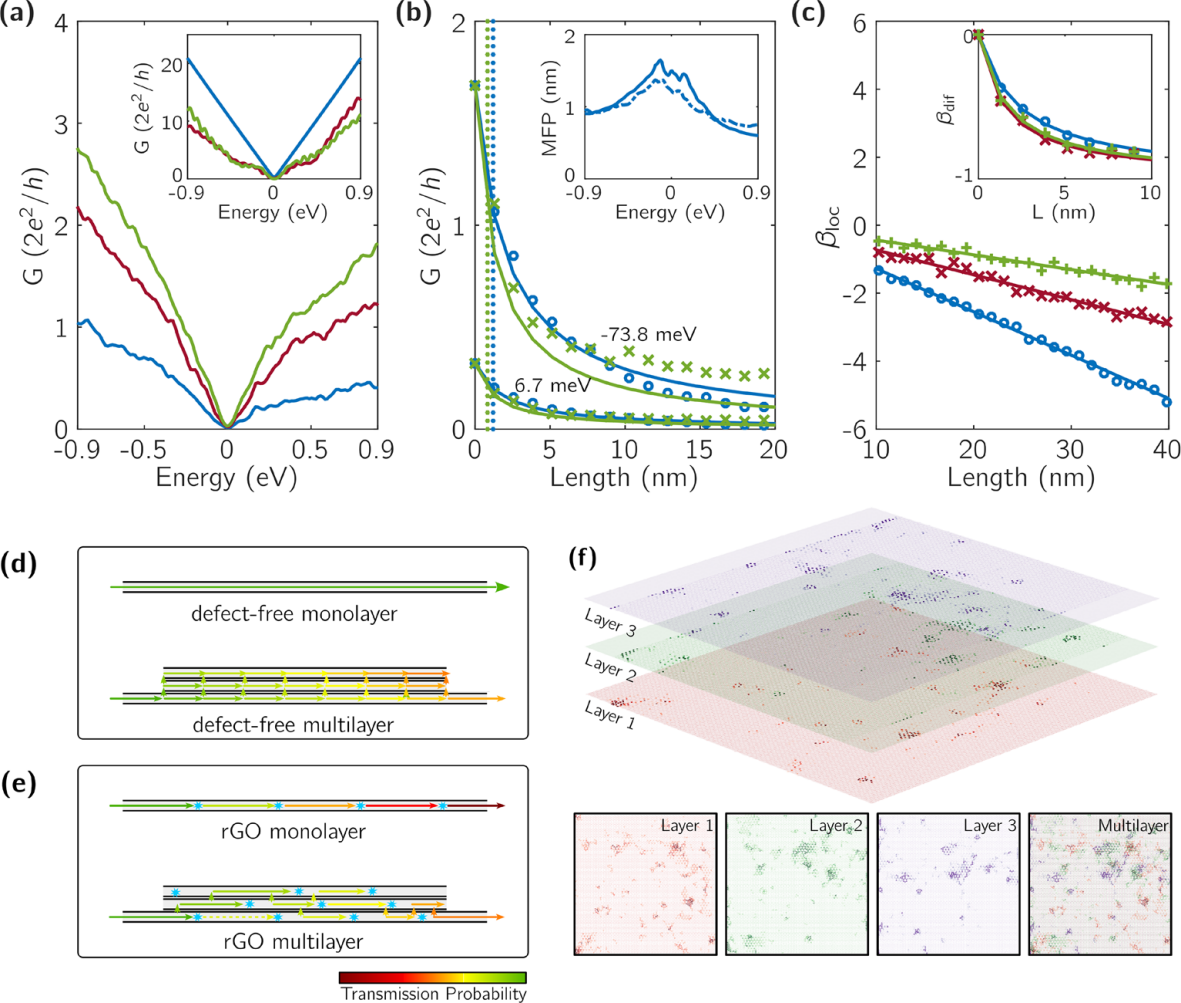
(a) Electronic conductance of mono/bi/trilayer rGO devices (blue, red, green curves, respectively) as a function of energy. The conductance is found to increase with the number of rGO layers, in contrast with stack of pristine graphene (inset). (b) Electronic conductance of mono- and trilayered rGO systems for *E* = 6.7 meV (lower region) and *E* = −73.8 meV (upper region) estimated by LB (markers) and eq 2 (solid lines). Mean free paths used in eq 2 are determined by using KG method. Diffusive versus localized regimes in multilayered rGO devices are distinguished by referring to the β-function (c). Transport regimes in pristine and defective stacks are illustrated in (d,e).(f) Local density of states (LDOS) around the Fermi level in multilayer rGO is shown for a relatively smaller sample. LDOS for individual layers and the multilayer are shown in the below panels. Red, green, and blue correspond to individual layers from bottom to top.

In sharp contrast, in strongly disordered rGO systems, the electronic behavior is opposite, in the sense that the higher the number of added layers on the stack, the larger the conductance (Figure 2a, main frame). This counterintuitive result suggests that the interlayer hopping is opening more conductive channels once the localization length of the bottom layer is short enough due to strong in-plane disorder. The two opposite roles played by interlayer coupling in defect-free and defective multilayers are pictured in Figure 2d,e.

To better understand the electronic transport mechanisms in mono- and multilayered rGO systems, we perform a scaling study of the conductance to better differentiate between diffusive and localized regimes. Figure 2b shows results for the mono- to multilayered rGO systems presenting the same defect density. Clear evidence for different scaling behaviors is observed (note that conductance values from LB simulations are marked with circles (monolayer) and crosses (trilayer)). In the diffusion regime, the conductance dependence with the length is theoretically expected to scale as a function of the mean-free-path () and the defect-free conductance (*G*_Graphene_) of the system (at a given energy) following
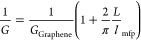
2where *L* is device length.^23^

For short enough channel length, the computed conductance scaling is indeed well described by the diffusive formula (eq 2), allowing the extraction of  from simulations. For the chosen disorder features,  in the range of 1–2 nm is obtained (Figure 2b, inset), regardless of the chosen transport formalism. As the number of layers is increased,  decreases slightly (Figure S5). We note that we report here the scaling behavior of *G* values for two selected energies (6.7 meV and −73.8 meV), but the trends are similar at all energies around the charge neutrality point (CNP).

We further observe that for channel length *L* > 10 nm, the conductance values for the monolayer are below the diffusion curve (which are plotted by solid lines). This pinpoints the onset of localization effects. However, for the trilayer case, a puzzling sudden change of the conductance behavior is seen for a channel length of 10 nm. To further substantiate this striking difference of the conductance scaling between monolayer and multilayered stack, we investigate the localization regime and capture quantitative information about the localization lengths. LB simulations are performed on rGO systems with long channel lengths and using the scaling function defined as β = ∂ ln *G*/∂ ln *L*.^28^ Substituting the corresponding expressions for diffusion and localization, β can be written as  in the diffusive regime, whereas  in the localization regime with  being the localization length. Both scaling functions (β_dif_ and β_loc_) are presented in Figure 2c versus the device length. The solid curves represent predictions of the aforementioned analytical formulas, whereas the markers are directly obtained from the simulation (for an energy of 6 meV) without referring to  or , since the scaling functions can be expressed as β_dif_ = *G*/*G*_Graphene_ – 1 and β_loc_ ∝ ln *G*.

On one hand, for the short-channel rGO devices, the diffusive regime is confirmed since the 1/*L* behavior is observed when using  (see Figure 2c, inset). On the other hand, localization regime is obtained for longer-channel rGO devices, and the evaluated localization lengths varies with the number of layers: 7.8 nm for monolayer, 13.7 nm for bilayer, and 22.9 nm for trilayer. Thus, when increasing the number of layers, hopping transport gives rise to larger transmission amplitudes compared to diffusion because of the enhancement of , in very good agreement with the experimental findings as shown in Figure 3.

**Figure 3:**
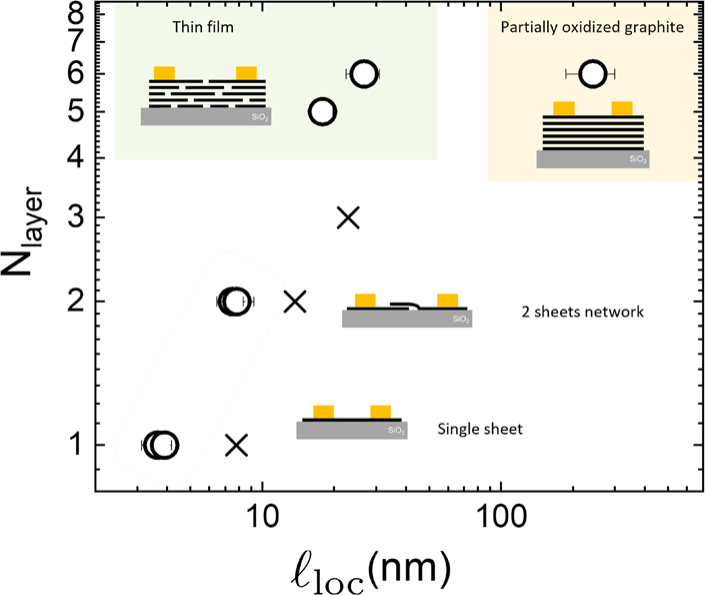
Localization lengths for varying numbers of layers. Circles represent experimental values, crosses are simulation results. *N*_layer_ ≤ 6 stands for randomly stacked rGO sheets, and the last data point is for partially oxidized graphite. Experimental  values are calculated using different dielectric constants. In the case of a single or a few stacked layers, we estimate ϵ_*r*_ = 2.5 from the average of the dielectric constant of the substrate (SiO_2_ = 3.9) and vacuum/air (= 1),^26^ while for RGO thin film we assume ϵ_*r*_ = 3.5,^27^ and ϵ_*r*_ = 15 for partially oxidized graphite.

Such behavior agrees well with experimental findings achieved on rGO devices with different numbers of layers (*N*_layer_) and similar chemical structure (i.e., sp^2^ content 96 ± 1%).^5^Figure 3 collects the results obtained comparing seven different systems ranging from the single nanosheet (*N*_layer_ = 1) to a flake of partially oxidized graphite (*N*_layer_ = 6). Differently, all the other devices (2 ≤ *N*_layer_ ≤ 6) are assemblies of rGO sheets randomly stacked. According with the results reported in ref (5), all six devices reveal ES-VRH transport mechanisms at low temperatures (10 K < *T* < 100 K). Thus, the corresponding localization length () is calculated using the temperature-dependent electrical resistivity curves  where ρ_0,VRH_ is a prefactor for resistivity, ϵ_r_ is the relative dielectric constant of the material, and *A* = 2.8*e*^2^/4*πϵ*_0_*k*_B_ = 0.021 μm^–1^ K^–1^ for a 2D system (see Supporting Information for more details). rGO devices have  values varying from ca. 4 to 30 nm, showing the same trend and the same order of magnitude when compared with the simulations. It is noteworthy to emphasize that in the case of partially oxidized graphite the corresponding  value is 1 order of magnitude larger amounting to 250 nm, clearly evidencing the combined role of the crystalline structure and the dielectric properties. Such aspects are out of the scope of this work, and some details related to the experimental setup and the device characterizations are reported in the Supporting Information.

The substantial increase in the localization lengths is thus a key transport feature to distinguish between various multilayered rGO devices and which can be rationalized withing the variable range hopping (VRH) framework.^29^ Indeed, at zero temperature, the energy separation between two localized states should be very small to enable significant hopping, with probability proportional to e^–2*αR*^ (with 1/α being the attenuation length for the localized states and *R* being the spatial separation of states). The hopping probability between two states ψ_*A*_ and ψ_*B*_, which have similar energies and are localized at the bottom layer well-separated from each other, should thus be strongly enhanced if a third state ψ_*C*_ is localized at the upper layer and between ψ_*A*_ and ψ_*B*_. Therefore, layers with localized states have enhanced transmission when stacked. In Figure 2f, the local density of states (LDOS) is plotted for a trilayer rGO sample.^34^ It is clearly visible that states localized at different layers tend to fill the spatial gaps when they are superimposed. This can qualitatively explain the enhancement of  with number of layers.

The interlayer coupling thus plays a crucial role in multilayer transport. In low defect concentrations, it is a source of scatterings which impedes transport, whereas in highly defective rGO multilayers, it tends to promote longer state delocalization. The contact geometry is also a major factor in transport across the layers. To gain further information about the combined roles of interlayer coupling and contact geometry, we simulate a situation where the device is made from overlapping bilayer rGO in which the injection and collection takes place at different layers (inset of Figure 4). We compute the conductance with different overlap distances, *L*, which is also the length of the central region. As shown in Figure 4, the conductance rapidly increases with *L* at short distances because the overlap area enhances the probability for a carrier to diffuse from one layer to the other. Differently, for overlap distances longer than 10 nm, the conductance decays exponentially with *L*, indicating that the localization behavior prevails over the interlayer diffusion. The maximum conductance is achieved at around 7 nm, which marks the interlayer diffusion length (). We have checked the dependence of  in clean structures, and found very similar values, which proves that  is dictated by the strength of interlayer coupling and not by the disorder content. We can actually estimate a length scale for interlayer diffusion as *hv*_*F*_/γ_1_ = 11.5 nm, where the Fermi velocity is *v*_*F*_ = 10^6^ m/s and the interlayer coupling strength is γ_1_ = 0.36 eV. The comparison  in the simulated structures is the reason behind the fact that  is weakly affected from the increase in the number of layers. On the other hand, since  is comparable with the monolayer localization length, there is room for localized carriers to further spread over the neighboring layers. To clarify the effect of contact geometry on transport, we have considered the electrodes with the same number of layers as in the central region. In this geometry, as expected, the conductance increases with the number of layers in both clean and defective samples, but the behavior of neither  nor  is considerably affected.

**Figure 4:**
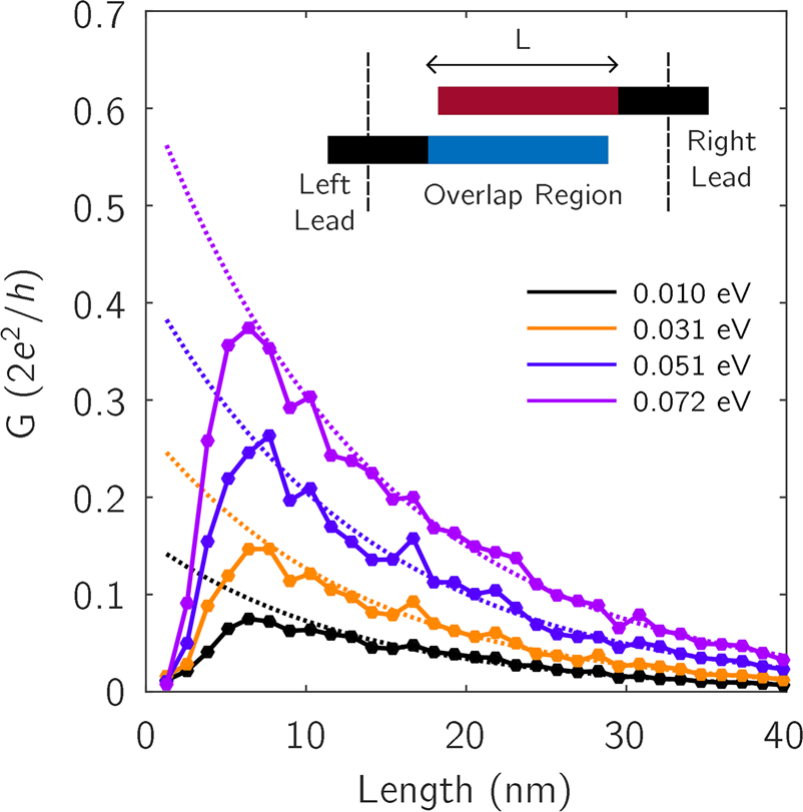
Conductance as a function of overlap length in bilayer reduced graphene oxide when the electrodes are connected to different layers. Dotted curves represent exponential decay.

Finally, based on these findings, the scaling of the resistivity with the number of layers can be expressed quantitatively. In highly defective rGO multilayers  changes only slightly with the number of layers, but  increases linearly with *N*_layer_. Using the fact that  is proportional to the number of transmission channels and ,^30,31^ one can approximate the transmission probability across multilayer rGO in terms of that of the monolayer as . Correspondingly, the resistivities (ρ) satisfy the following relation
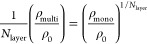
3where ρ_0_ = *hA*_mono_/2*e*^2^*L* is a system-wide constant *A*_mono_ being the cross section area for a monolayer. Specifically, we predict a linear dependence of ln(ρ_multi_/*N*_layer_) ∼ 1/*N*_layer_ for film thickness larger than the mean free path. For the sake of comparison, we analyze a data set of 11 rGO devices with similar chemical structure (i.e., sp^2^ content = 96 ± 1%) and film thickness ranging from 2 to 13 nm, i.e., 5 < *N*_layer_ < 35. Note that we do not need to assume any values for *A*_mono_ and ρ_0_ or measure them, but they are used in order to relate resistivity and conductance so as to compare experimentally measured values with simulated ones through the scaling relation (eq 3).

Figure 5 shows the correlation plot ln(ρ_multi_/*N*_layer_) vs 1/*N*_layer_ for each device displaying the resistivity values acquired at different temperatures: 5, 100, and 300 K; the lowest, an intermediate, and the highest measured, respectively. A total of data sets corresponding to 43 different temperatures were analyzed, and the remaining 40 curves—not depicted in the figure—are included between the two curves acquired at 5 and 300 K (dashed area). In all the cases we observe a linear trend *y* = *m*·*x* + *q* in excellent agreement with eq 3. Moreover, we determined that the slope *m* = ln(ρ_mono_/ρ_0_) decreases with increasing temperature, while the *y*-intercept (*q*) is a constant value corresponding to ρ_0_ = exp(*q*) = (2.1 ± 0.2) × 10^–7^ Ω·m. Similarly, a linear behavior is achieved in the case of rGO devices with lower amount of the aromatic content (77% and 86%, see Figure S9) where the resistivity values increase with the oxidation degree, as expected. To summarize the experimental findings, ρ_mono_ is the single-layer resistivity (temperature-dependent), while ρ_0_ does not depend on the temperature, being therefore a kind of resistivity scaling factor only depending on the aromatic content of the device.

**Figure 5:**
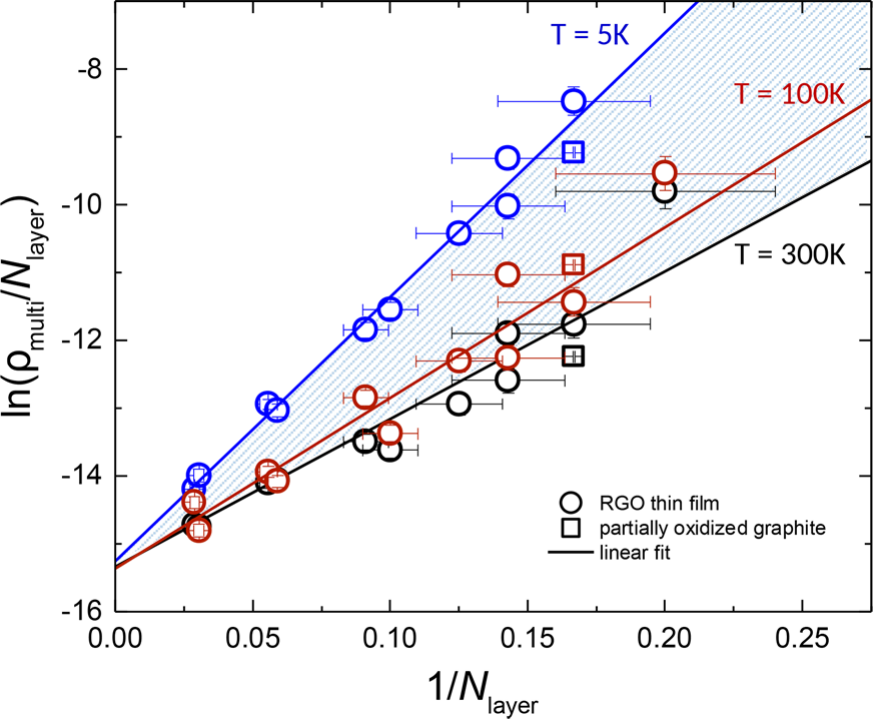
Scaling of multilayer resistivity with the number of layers is plotted as ln(ρ_multi_/*N*_layer_) versus 1/*N*_layer_. Experimental data of multilayer rGO (circles for devices reported in ref (5) and square for partially oxidized graphite) acquired at different temperatures show linear dependence, in good agreement with the theoretical prediction for scaling (cf., eq 3). All the linear fitting curves calculated at different temperatures are included between the two curves acquired at 5 and 300 K (dashed area).

## Conclusion

We have reported quantum simulations on realistic models of multilayered rGO which reveal the complex interplay between disorder and interlayer interactions in dictating the dominant transport mechanism. Depending on the concentration of defects, multilayer interactions can enhance or suppress the system conductance, which results from the competition between the mean free path  and the interlayer diffusion length . When about 5% of the carbon atoms are involved in defected regions,  becomes much longer than . In that case, intralayer scattering largely dominates over interlayer diffusion, leading to a weak dependence of  on *N*_layer_.

On the other hand, , so that a localized state in one of the layers has enough extension for tunneling to an adjacent one. If  was much larger than , the tunneling rates would be much smaller. Once , tunneling rates are appreciable and charge delocalization is promoted. While  is weakly dependent on *N*_layer_,  increases with *N*_layer_ as expected from a generalization of the Thouless relationship for one-dimensional conductors. This unprecedented interplay between transport length scales is a specific result of 2D layered nature of the multilayer rGO systems. Such a mechanism enables hopping transport to overcome the diffusion limit, which is usually the upper bound in bulk systems. Our theoretical analysis enables us to derive a novel scaling rule, which is in perfect agreement with experimental data at various temperatures and consistent with the Thouless relationship. The fundamental findings of this study are not limited to multilayered reduced graphene oxide but could find applications in other two-dimensional stacks as well.

## Supporting Information Available

Kubo-Greenwood and Landauer-Büttiker methods; simulated structures; system setups; tight-binding parameters; supplementary mean-free-path plots; results with lower defect concentrations; supplementary LDOS plots; temperature dependence of electrical resistivity ρ(*T*) ; Efros–Shklovskii variable range hopping model; details of electrical resistivity measurements; table of experimental parameters of all the studied devices (PDF)
