# Supplementary material for: Toward Optimized Charge Transport in Multilayer Reduced Graphene Oxides
Source: Nano Lett. 2022 Mar 1;22(6):2202–8. doi: 10.1021/acs.nanolett.1c03883 (PMC8949764; doi:10.1021/acs.nanolett.1c03883)
Supplement: Supplementary file 1 — nl1c03883_si_001.pdf [file nl1c03883_si_001.pdf]

Supporting Information

# Towards Optimized Charge Transport in Multilayer Reduced Graphene Oxides

Mustafa Neşet Çınar, Aleandro Antidormi, Viet-Hung Nguyen, Alessandro Kovtun, Samuel Lara Avila, Andrea Liscio, Jean-Christophe Charlier, Stephan Roche, Hâldun Sevinçli

E-mail: stephan.roche@icn2.cat; haldunsevincli@iyte.edu.tr

## Kubo-Greenwood and Landauer-Büttiker methods

An efficient linear scaling approach[25] is used in Kubo transport to estimate the energy- and time-dependent mean squared displacement of the wave-packet that spreads into the investigated atomic structure,

$$\Delta X^2(E,t) = \frac{\mathrm{Tr}\left[\delta(E-\hat{H})|\hat{X}(t)-\hat{X}(0)|^2\right]}{g(E)}, \tag{S1}$$

where $g(E) = \mathrm{Tr}[\delta(E-\hat{H})]$ is the density at energy $E$. The time-dependent semiclassical diffusion coefficient $D(E,t) = \frac{\partial}{\partial t}\Delta X^2(E,t)$ and its asymptotic limit $\tilde{D}(E)$ can then be calculated, allowing for the computation of both the electron conductivity and the mean free path as $\sigma(E) = e^2 g(E)\tilde{D}(E)$ and $\ell_{\mathrm{mfp}}(E) = 2\tilde{D}(E)/v_F(E)$, respectively (with $v_F(E)$ being the carrier velocity). The conductivity values in rGO systems have been averaged over 10 different randomly chosen initial wave packets, and the calculation of the mean-square displacement was carried out through an efficient decomposition in terms of Chebyshev polynomials, with 5000 moments. Note that periodic boundary conditions are employed in

both longitudinal and transverse directions.

Concerning the transport simulations performed using the Landauer-Büttiker technique, the rGO system is partitioned into three regions, namely the left and right electrodes (leads) and the central region. The leads are modeled as scattering free regions made up of the same ideal material. The Green function for the central region is calculated as $\mathcal{G}(E) = [(E + i0^+)I - H_C - \Sigma]^{-1}$, where $I$ is identity matrix, $H_C$ is the Hamiltonian matrix for the central region, and the self-energy term includes effects of the left and right reservoirs as $\Sigma = \Sigma_L + \Sigma_R$. In this work, systems containing as many as $10^6$ atoms have been simulated, for which efficient decimation algorithms are implemented.[24] The transmission amplitude is obtained from $\mathcal{T}(E) = \mathrm{Tr}\left[\Gamma_L \mathcal{G} \Gamma_R \mathcal{G}^\dagger\right]$, where $\Gamma_{L(R)} = i[\Sigma_{L(R)} - \Sigma^\dagger_{L(R)}]$ are the left (right) broadening matrices. Conductance values are calculated using the Landauer formula,

$$G = \frac{2e^2}{h} \int \left( -\frac{\partial f_{FD}(E, \mu, T)}{\partial E} \right) \mathcal{T}(E) dE, \qquad \text{(S2)}$$

where $e$ is electron charge, $f_{FD}$ is Fermi-Dirac distribution function, $T$ is temperature (herewith 100 K) and $\mathcal{T}(E)$ is the transmission probability for a given energy $E$. Since low-energy properties are only of interest, transmission coefficients are integrated over 40 $k$-points in the transverse direction in order to reach sufficiently accurate energy resolution. At last, a geometric average on the transmission function over an ensemble of 20 samples is applied to overcome the sample size effects.

# Supplementary details on simulated structures

The types and amounts of defects are deduced from MD simulations replicating the thermal annealing of GO on a computer. In particular, following the protocol in Ref. 6, several atomistic samples of GO have been generated with a total number of atoms as large as 10000 and an initial oxygen concentration of 35%. The thermal reduction of the systems has been simulated for different annealing temperatures and a statistical analysis of the chemical and morphological properties of the resulting rGO samples has been performed. In the table below, a summary of the values of the most relevant chemical species observed in the the samples after reduction is given. At the annealing temperature of 900oC, a final concentration of oxidizing agents of 5% has been found, in extremely good agreement with the experimental samples. From a detailed exploration of the atomistic structures, the concentration and type of defects has been derived and used to model rGO samples for transport calculations.

Table 1: Concentration of different chemical species observed in the atomistic samples of rGO after the reduction process at different annealing temperatures.

| | C $sp^2$ (%) | C $sp^3$ (%) | C-OH (%) | C-O-C (%) | C=O (%) | O-C=O (%) | O/CFIT |
|---|---|---|---|---|---|---|---|
| GO | 32.9 | 11.5 | 3.0 | 43 | 6.5 | 3.0 | 0.35 (imposed) |
| rGO 300 °C | 75.4 | 10.5 | 2.9 | 3.9 | 4.8 | 2.5 | 0.154 |
| rGO 600 °C | 83.5 | 8.8 | 1.1 | 3.7 | 1.1 | 1.8 | 0.11 |
| rGO 900 °C | 94.1 | 3.6 | 0.3 | 0.8 | 0.6 | 0.6 | 0.05 |

# Supplementary details regarding tight-binding parameters

As explained in the main text, while the change in C-C bond length is modeled by the distance dependence of hopping energies, the local doping due to the localized states induced by defects and impurities are included by adding on-site energies to C-atoms surrounding their position. These on-site energies have the following common form

- $\varepsilon_D$ applied to C atoms directly connected to impurities/defects
- $\varepsilon_n$ applied to other surrounding C atoms

$$\varepsilon_n = \frac{\varepsilon_0}{1 + (d_n/\lambda_D)^\kappa} \tag{S3}$$

where $d_n$ is the distance from the $n^{th}$ C atom to the considered impurity, $\varepsilon_0$ is the maximum value of $\varepsilon_n$, $\lambda_D$ is the decay length and the number $\kappa$ is determined depending on the defect/impurity types.

| | | | | | |
|---|---|---|---|---|---|
| Oxygen impurity | $\lambda_D = 1.0$ Å | $\kappa = 3$ | $\varepsilon_0 = 1.6$ eV | $\varepsilon_D = -28.0$ eV | |
| OH group | $\lambda_D = 1.0$ Å | $\kappa = 3$ | $\varepsilon_0 = 1.8$ eV | $\varepsilon_D = -28.0$ eV | |
| 585 defect | $\lambda_D = 5.0$ Å | $\kappa = 5$ | $\varepsilon_0 = 1.0$ eV | $\varepsilon_D = -1.3$ eV | |
| Stone-Waled defect | $\lambda_D = 1.6$ Å | $\kappa = 5$ | $\varepsilon_0 = -1.5$ eV | $\varepsilon_D = -1.2$ eV | |
| 555-777 defect | $\lambda_D = 8.0$ Å | $\kappa = 5$ | $\varepsilon_0 = 0.4$ eV | $\varepsilon_{D1} = -1.8$ eV | $\varepsilon_{D2} = -0.5$ eV |

The electronic band structures obtained using the proposed tight binding Hamiltonians are presented in Figs. S2 and S3. Indeed, our proposed tight-binding models reproduce well the low energy bands, compared to the DFT results.

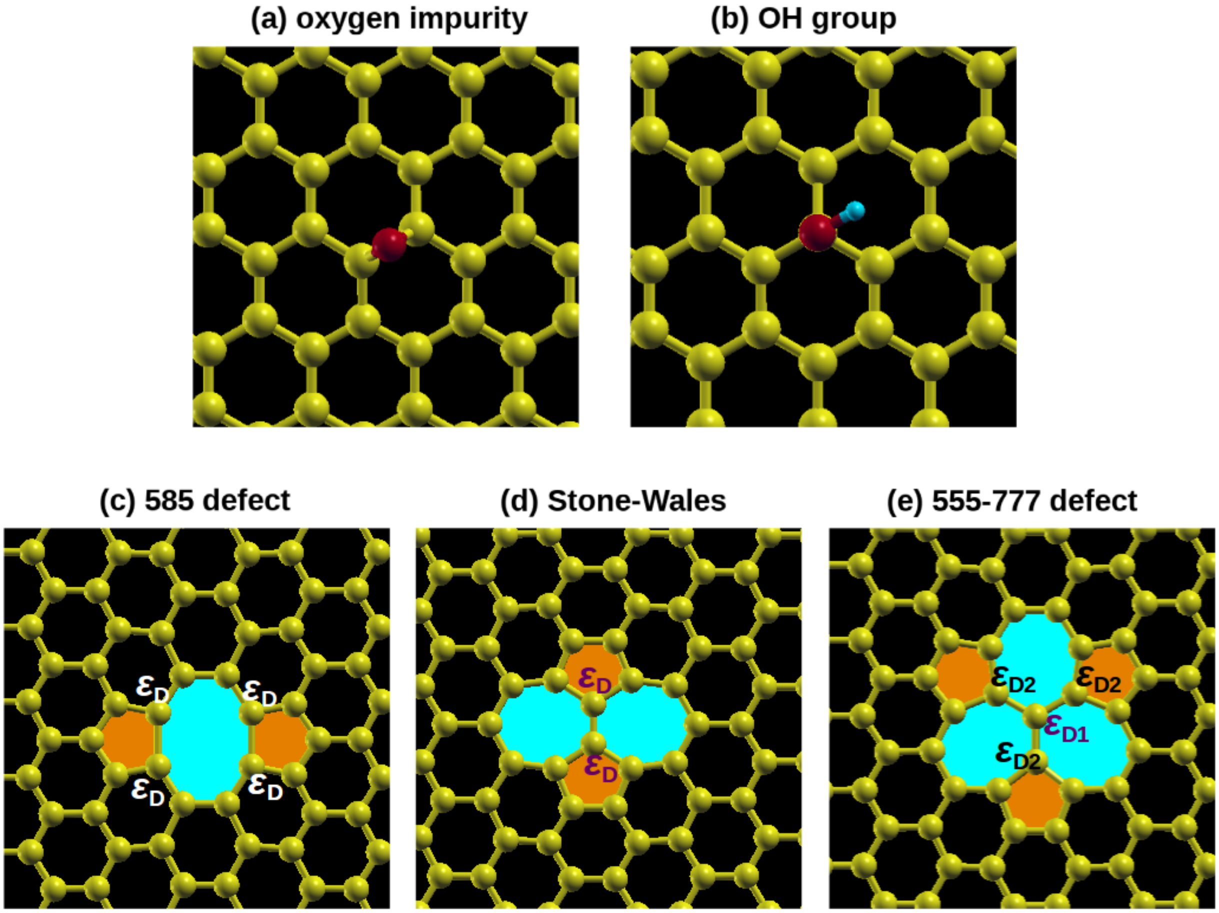


Figure S1: Defects and impurities investigated in this work.

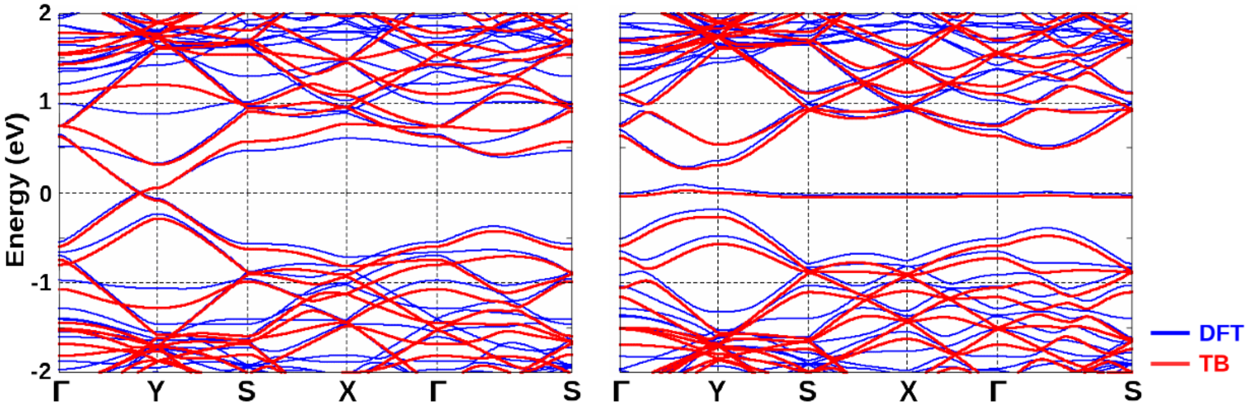


Figure S2: The electronic band structure of graphene with oxygen impurity (left) and OH group (right). TB calculations fit to the DFT results.

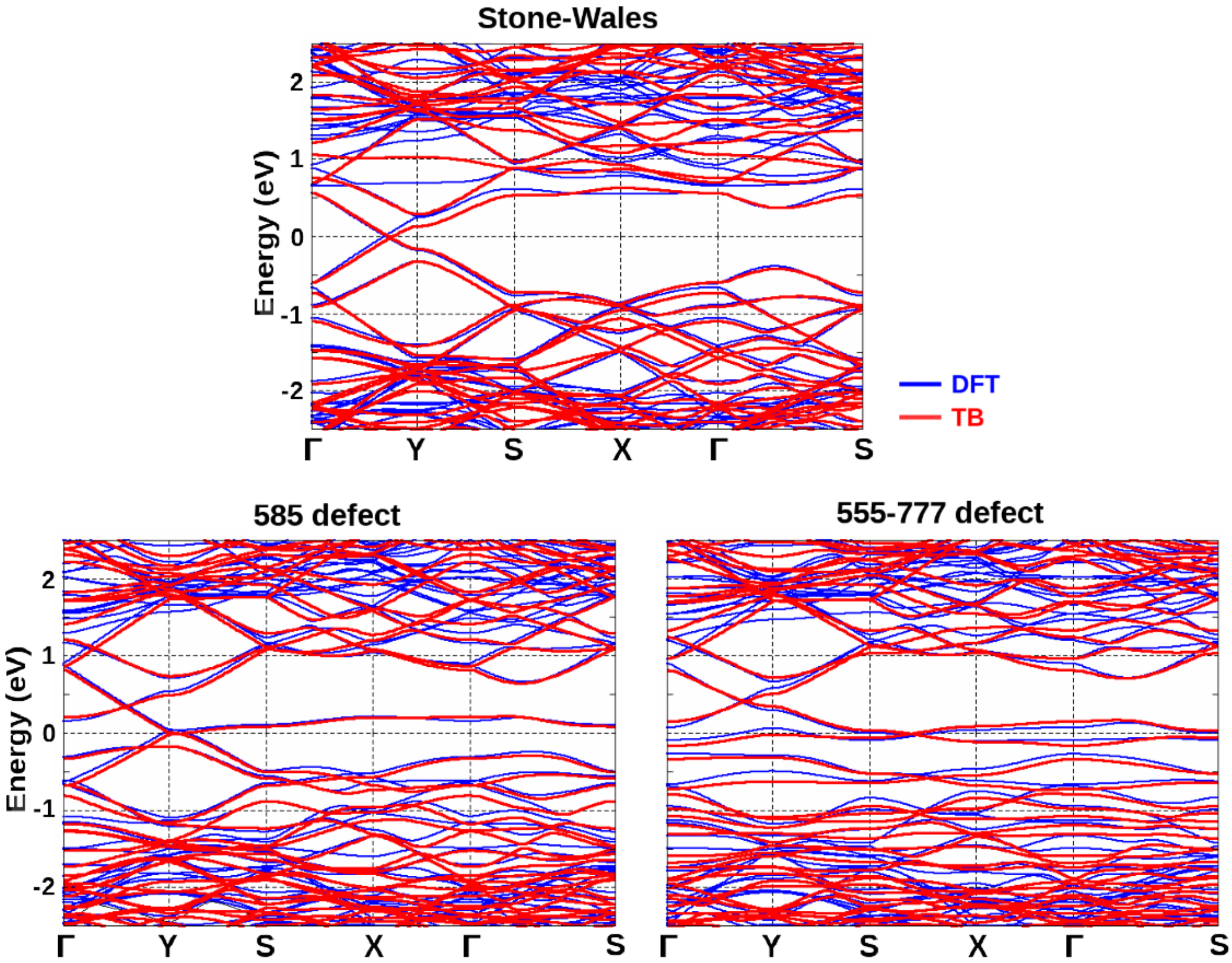


Figure S3: The electronic bandstructure of graphene with structural defects. TB calculations fit to the DFT results.

Other sophisticated models (i.e., larger distance neighbor as well as Slater-Koster like models) generally present a disadvantage that a large number of adjusted parameters are required to model accurately the considered defective systems. This disadvantage also gives rise to some difficulties for the implementation of transport calculations in the large scale devices while the accuracy is not significantly improved. In Fig. S4 a comparison of the computed electronic band strucures of bilayer graphene obtained using our used model and Slater-Koster like models in Ref. 32 that has been shown to compute well the electronic structure of both Bernal stacking and twisted bilayer graphene systems.

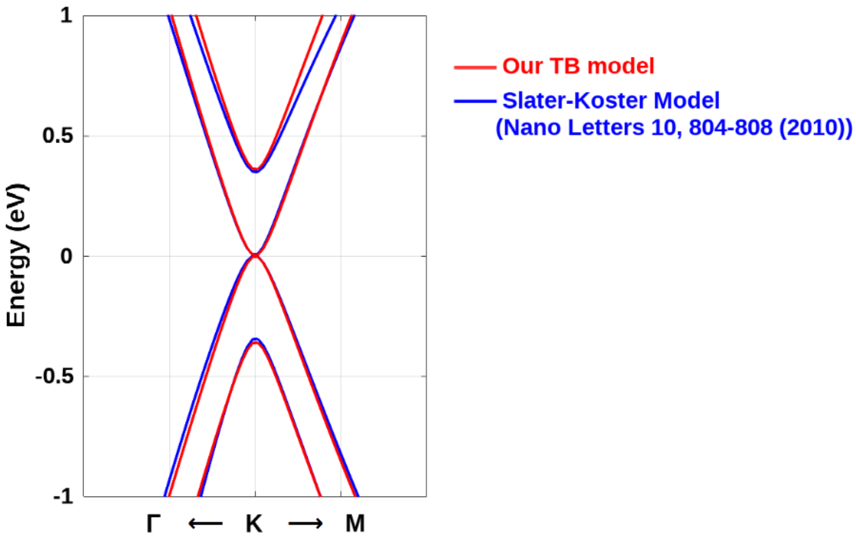


Figure S4: Comparison of the electronic bands of bilayer graphene obtained using our TB Hamiltonian and Slater-Koster like models in Nano Lett. 10, 804–808 (2010).

# Note on parametrization and system setups

The tight-binding parametrization for structural defects involves modifying onsite energies of $sp^2$ orbitals by a value which decays exponentially with the distance, and onsite energies due to defects surrounding a particular atom are taken additive. For Landuer-Büttiker calculations, we also introduced defect-free buffers of 2.57 nm in length between the scattering region and the leads to saturate the effects due to the onsite energies (see Fig.1 right panel), and cutoff radius of 2.57 nm was used as the range of the modification. For the scaling analysis, the central transport channel is lengthened by adding 1.284 nm-length blocks (which corresponds to a mesh resolution of the same length).

## Supplementary mean-free-path plots

Mean-free-paths as obtained from Landauer-Büttiker and Kubo-Greenwood simulations with changing layer thickness are shown for comparison in Figure S5. The agreement between two methodologies is remarkable.

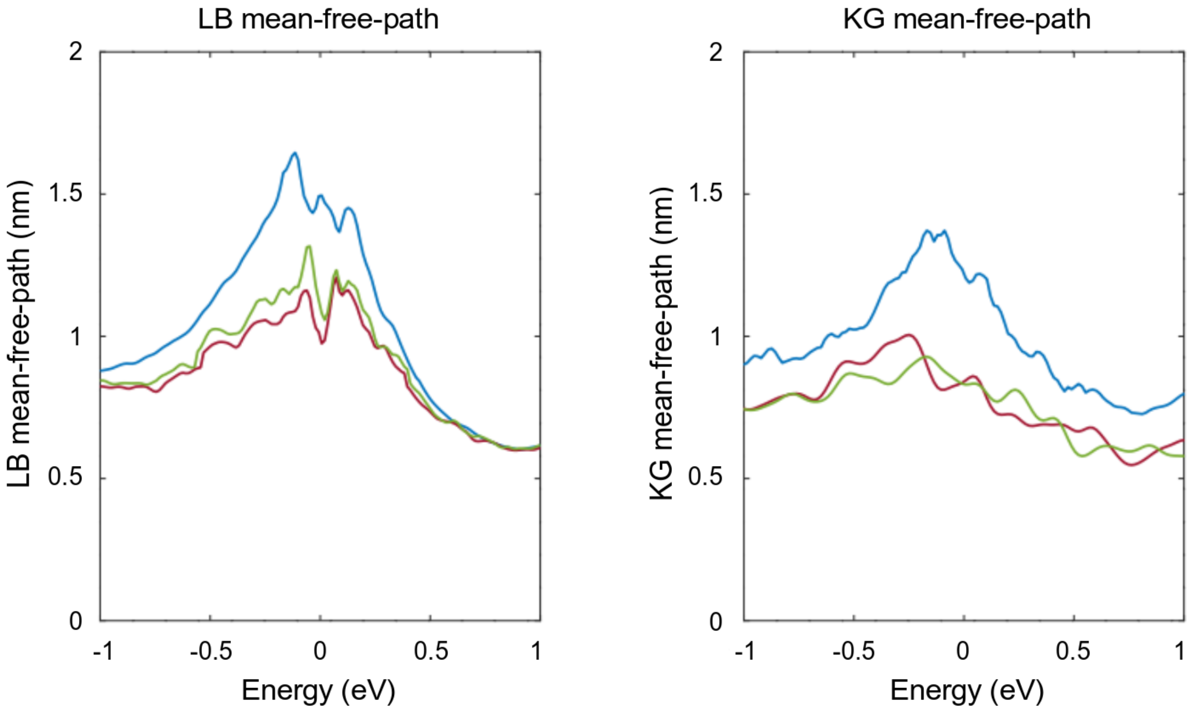


Figure S5: Mean-free-paths for different numbers of layers as obtained from NEGF and KG simulations are shown. Blue, red and green curves represent mono/bi/tri-layer rGO systems.

# Supplementary results with lower defect concentrations

We have shown in the main text that interlayer coupling affects charge transport of defect-free and defective systems in opposite ways. Namely, in defect-free systems conductance is reduced with the number of layers, whereas it is enhanced in rGO, which contains 95% $sp^2$ carbon. Reducing the amount of impurities, it is possible to observe the transition. In Fig. S6, defect concentration is 10 times lower than those in the main text (99.5% $sp^2$ carbon), where monolayer is observed to have the highest transmission values around the charge neutrality point.

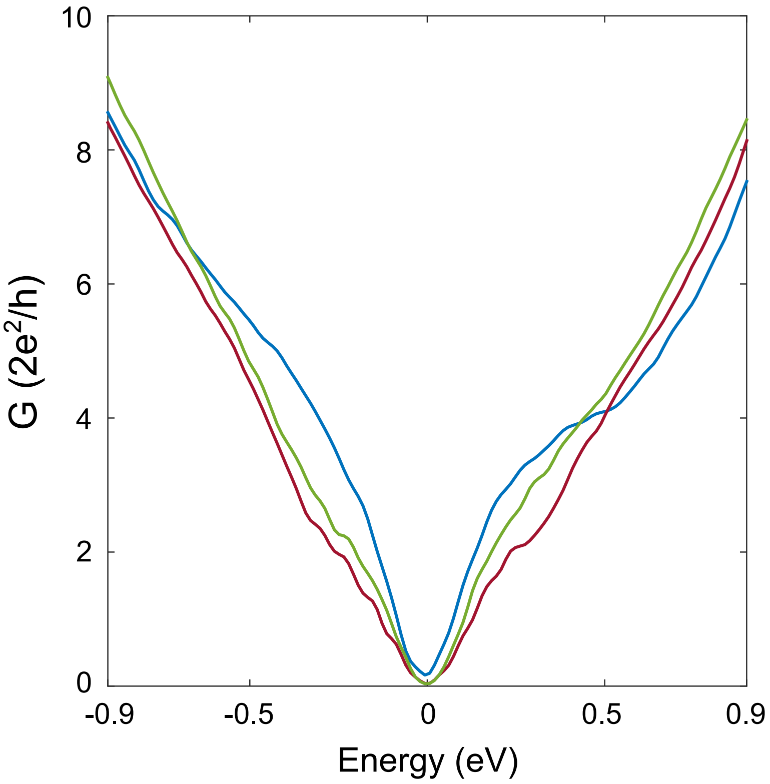


Figure S6: Transmission spectrum through rGO with low concentration of imperfections. The device sizes are the same with those in the main text, impurity concentration is 10 times lower with 99.5% $sp^2$ ratio. Blue, red and green curves correspond to mono/bi/trilayer rGO, respectively.

# Supplementary LDOS plots

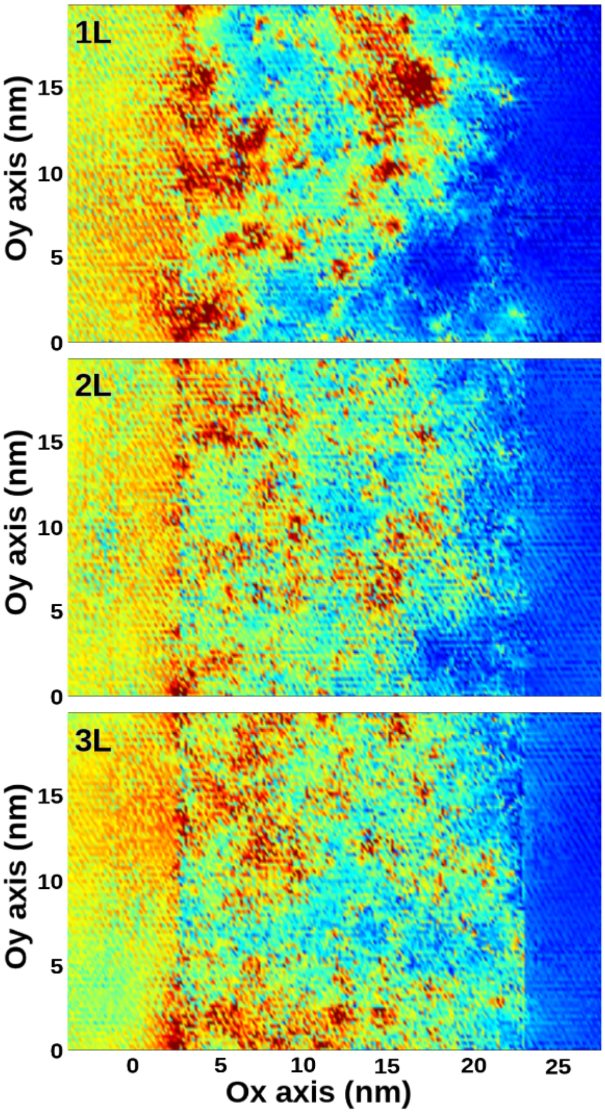


Figure S7: Left injected LDOS plots for mono/bi/tri-layer rGO. High and low LODS values are distinguihed on the left/right electrodes. Localization is more pronounced in monolayer, whereas the carrier density more dispersed in trilayer sample.

Using the Green's function method, we could compute the left (right, respectively) injected LDOS,[33] reflecting the propagation of electrons from left to right (right to left, respectively) electrodes. In particular, the left-injected LDOS is given by

$$LDOS_L = \frac{G\Gamma_L G^\dagger}{2\pi}. \tag{S4}$$

LDOS in the mulitlayer zones are averaged over the layers to show the contributions from all layers. The decay of the presented left-injected LDOS along the Ox axis in Fig.S6 is essentially due to scatterings with defects/impurities, manifesting as the electronic localization in the device region. In the monolayer case, charge localization is more pronounced than bilayer and trilayer systems, in agreement with Fig. 2f. Importantly, it is shown that the improved propagation of electrons from left to right electrodes is obtained when increasing number of graphene layers, thus illustrating the transport properties discussed in the main text.

# Temperature dependence of electrical resistivity $\rho(T)$, Efros-Shklovskii variable range hopping model

At low temperature, charge transport in graphene-based materials is typically occurring via charge hopping in a disorder-broadened density of states near the Fermi level $g(E_F)$.[34] In the Ohmic regime, the resistivity is tipycally modelled by a stretched exponential behavior:

$$\rho(T) = \rho_{0,VRH} \exp\left(\frac{T_0}{T}\right)^{\beta}, \tag{S5}$$

where $\rho_{0,VRH}$ is a prefactor and $\beta$ is a characteristic exponent. $T_0$ represents a characteristic temperature correlated to the localization length ($\ell_{\text{loc}}$), the higher the first one, the lower the latter. The $\ell_{\text{loc}}$ is defined as the average spatial extension of the charge carrier wave function: the lower the $T_0$, the larger the $\ell_{\text{loc}}$.

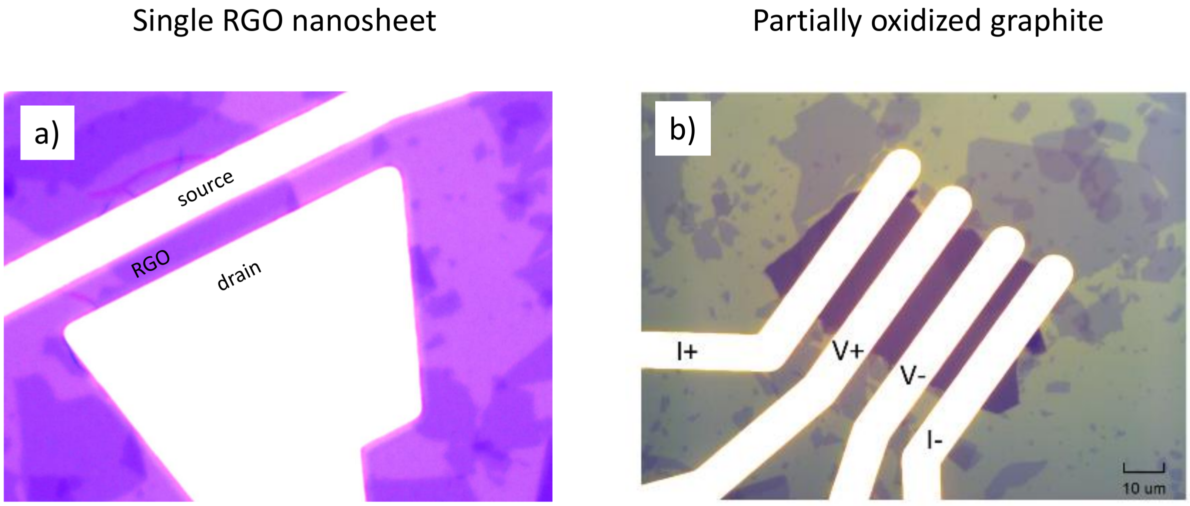


Figure S8: Optical images of devices fabricated using (a) single rGO nanosheet and (b) partially oxidized graphite. Samples thickness were measured by atomic force microscopy (AFM) and the graphitic structure of (b) was confirmed by Raman measurements.

The analytic expression reported in Eqn. S5 is quite general depending on the model commonly referred as variable range hopping (VRH). The stretching exponent $\beta$ is strongly dependent on the shape of $g(E_F)$, e.g. when the density of states is constant (Mott-VRH

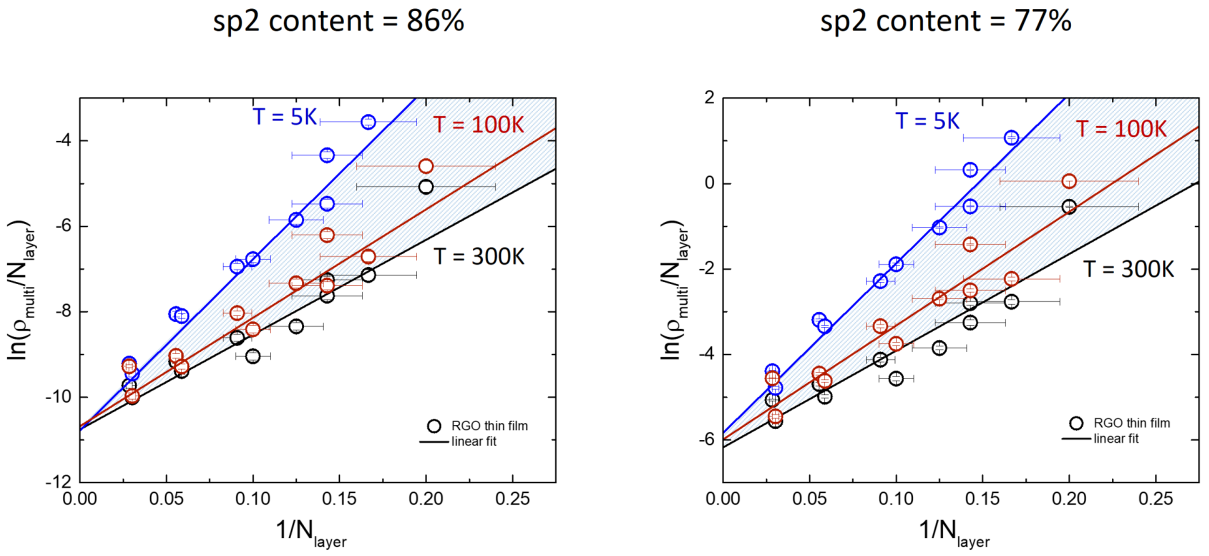


Figure S9: Correlation plots. Experimental data of multilayer RGO (circles for devices reported in Ref. 5 and acquired at different temperatures. Samples with both $sp^2$ contents (86% and 77%) show linear dependence, in good agreement with the theoretical prediction for scaling (cf. Eqn. 3 in the main text). All the linear fitting curves calculated at different temperatures are included between the two curves acquired at 5 K and 300 K (dashed area).

model),[35] the $\beta$ value directly depends on the system's dimension $(D)$ with the form $\beta = 1/(D+1)$. Reduced graphene oxide thin films show the presence of a gap at the Fermi level due to the Coulomb interaction between the occupied, excited state above $E_F$ and the hole left by the same electron below $E_F$. This case is described by the so called Efros Shklovskii model (ES-VRH)[36] with characteristic exponent of Eqn. S5 $\beta = 1/2$, which does not depend on the system dimensionality. The characteristic temperature $T_0$ for 2D materials is given by

$$T_0 = \frac{2.8e^2}{4\pi\epsilon_0\epsilon_r k_B \ell_{\mathrm{loc}}} = \frac{1}{A\epsilon_r \ell_{\mathrm{loc}}}, \tag{S6}$$

where $e$ is the elementary charge, $\epsilon_0$ and $\epsilon_r$ represent the vacuum permittivity and the relative permittivity of the material and $k_B$ is the Boltzmann constant. For the sake of simplicity, all the universal constants are collected by the parameter $A = 0.021\mu\mathrm{m}^{-1}\mathrm{K}^{-1}$. Combining

Eqns. S5 and S6, we obtain the mathematical expression reported in the main text,

$$\rho(T) = \rho_0 \exp\left(\frac{1}{A\epsilon_r \ell_{\mathrm{loc}} T}\right)^{1/2}. \tag{S7}$$

# Electrical resistivity measurements $\rho(T)$

Single rGO nanosheet and partially oxidized graphite were prepared by thermal annealing ($T_{\text{ann}} = 900^o$C) of GO and oxidized nanographite, respectively, deposited on clean $SiO_2$/Si substrates (2,000 rpm for 60s).

The micrometric electrodes were lithographically patterned to characterize the electrical transport across a limited number of overlapping flakes. Lithography was carried out by exposing a standard photoresist (AZ1505, Microchemicals) with the 405 nm laser of a Microtech laser writer. A 30-nm-thick Au film (without adhesion layer) was thermally evaporated onto the patterned photoresist and lift-off was carried out in warm acetone (40$^o$C).

The resistance vs temperature measurements were carried out with a Quantum Design Physical Properties Measurements System (PPMS), using an external Keithley 2636 Source-Meter. The resistance was measured in the temperature range between 300 K to 5 K with a slow ramp (1 K/min). The Ohmic behavior of the device was checked by the linearity of the I-V curves.

Typically, each acquired $\rho(T)$ curve corresponds to an array of >50,000 resistivity values at different temperatures. For sake of simplicity, such $\rho(T)$ curves were sampled at 43 temperature values with logarithmic steps, as reported in Fig. S10. Three values acquired at 5 K, 100 K and 300 K are reported in Figure 5 in the main text.

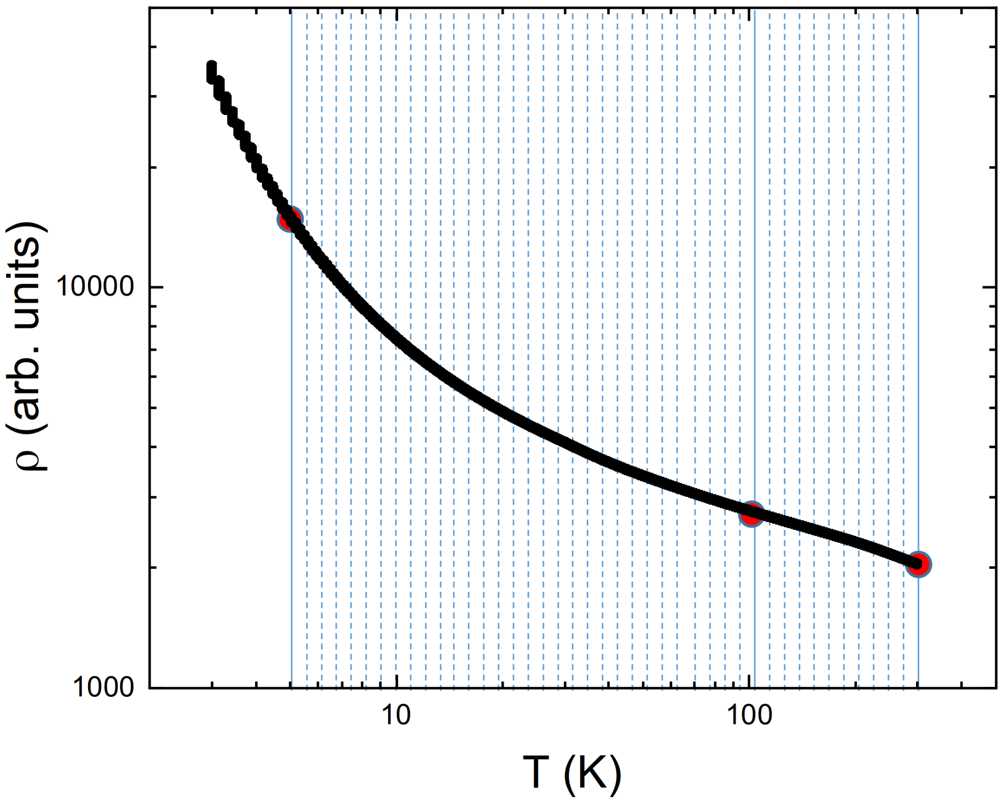


Figure S10: Example of measured $\rho(T)$ curve and sampled temperature values. Red circles correspond to the resistivity values depicted in the correlation plots.

Table 2: Summary of experimental parameters of all the studied devices

| sample | | $N_{\text{layer}}$ | $\xi$ (nm) | $\rho_{5K}$ | $\rho_{100K}$ | $\rho_{300K}$ |
|---|---|---|---|---|---|---|
| single RGO nanosheet | Device 28 [ref.5 main text] | 1 | 4.0±0.3 | | | |
| | | 1 | 3.7±0.4 | | | |
| Bi-layer RGO | Device 26 | 2 | 7.5±0.8 | | | |
| | Device 27 | 2 | 7.8±1.4 | | | |
| RGO thin film | Device 6 | 5±1 | 18±2 | – | $(3.6 \pm 12) \times 10^{-4}$ | $(2.8 \pm 0.9) \times 10^{-4}$ |
| | Device 1 | $6 \pm 1$ | $26 \pm 4$ | $(13 \pm 3) \times 10^{-4}$ | $(6.5 \pm 1.7) \times 10^{-5}$ | $(4.7 \pm 1.3) \times 10^{-4}$ |
| partially oxidized graphite | | $6 \pm 1$ | $243 \pm 56$ | $(5.9 \pm 1.3) \times 10^{-4}$ | $1.1 \pm 0.3) \times 10^{-4}$ | $(2.9 \pm 0.7) \times 10^{-5}$ |
| RGO thin film | Device 7 | $7 \pm 1$ | | $(3.1 \pm 0.7) \times 10^{-4}$ | $(3.4 \pm 0.8) \times 10^{-5}$ | $(2.4 \pm 0.6) \times 10^{-5}$ |
| | Device 17 | $7 \pm 1$ | | $(6.4 \pm 1.4) \times 10^{-4}$ | $(1.1 \pm 0.2) \times 10^{-4}$ | $(4.8 \pm 1.1) \times 10^{-4}$ |
| | Device 2 | $8 \pm 1$ | | $(2.4 \pm 0.5) \times 10^{-4}$ | $(3.7 \pm 0.7) \times 10^{-5}$ | $(1.9 \pm 0.4) \times 10^{-5}$ |
| | Device 8 | $10 \pm 1$ | | $(9.7 \pm 1.5) \times 10^{-5}$ | $(1.6 \pm 0.2) \times 10^{-5}$ | $(1.2 \pm 0.2) \times 10^{-5}$ |
| | Device 3 | $11 \pm 1$ | | $(7.9 \pm 1.2) \times 10^{-5}$ | $(2.9 \pm 0.4) \times 10^{-5}$ | $(1.5 \pm 0.2) \times 10^{-5}$ |
| | Device 9 | $17 \pm 1$ | | $(3.7 \pm 0.4) \times 10^{-5}$ | $(1.3 \pm 0.1) \times 10^{-5}$ | $(1.3 \pm 0.1) \times 10^{-5}$ |
| | Device 4 | $18 \pm 1$ | | $(4.3 \pm 0.4) \times 10^{-5}$ | $(1.6 \pm 0.2) \times 10^{-5}$ | $(1.4 \pm 0.1) \times 10^{-5}$ |
| | Device 10 | $33 \pm 2$ | | $(2.8 \pm 0.3) \times 10^{-5}$ | $(1.2 \pm 0.1) \times 10^{-5}$ | $(1.3 \pm 0.1) \times 10^{-5}$ |
| | Device 5 | $35 \pm 2$ | | $(2.4 \pm 0.2) \times 10^{-5}$ | $(2.0 \pm 0.2) \times 10^{-5}$ | $(1.4 \pm 0.2) \times 10^{-5}$ |
